# Influence of initial misdiagnosis on mortality in patients with bacteraemia: propensity score matching and propensity score weighting analyses

**DOI:** 10.1186/s12879-024-09299-9

**Published:** 2024-04-11

**Authors:** Anna M. Eikenboom, Merel M. C. Lambregts, Mark G. J. de Boer, Saskia le Cessie

**Affiliations:** 1 Department of Infectious Diseases, Leiden University Medical Centre (LUMC), Leiden, The Netherlands; 2 Department of Clinical Epidemiology, Leiden University Medical Centre (LUMC), Leiden, The Netherlands; 3 Department of Biomedical Data Sciences, Leiden University Medical Centre (LUMC), Leiden, The Netherlands

**Keywords:** Misdiagnosis, Propensity score matching, IPTW, Bacteraemia, Mortality

## Abstract

**Background:**

The diagnostic process is a key element of medicine but it is complex and prone to errors. Infectious diseases are one of the three categories of diseases in which diagnostic errors can be most harmful to patients. In this study we aimed to estimate the effect of initial misdiagnosis of the source of infection in patients with bacteraemia on 14 day mortality using propensity score methods to adjust for confounding.

**Methods:**

Data from a previously described longitudinal cohort of patients diagnosed with monobacterial bloodstream infection (BSI) at the Leiden University Medical Centre (LUMC) between 2013 and 2015 were used. Propensity score matching and inversed probability of treatment weighting (IPTW) were applied to correct for confounding. The average treatment effect on the treated (ATT), which in this study was the average effect of initial misdiagnosis on the misdiagnosed (AEMM), was estimated. Methodological issues that were encountered when applying propensity score methods were addressed by performing additional sensitivity analyses. Sensitivity analyses consisted of varying caliper in propensity score matching and using different truncated weights in inversed probability of treatment weighting.

**Results:**

Data of 887 patients were included in the study. Propensity scores ranged between 0.015 and 0.999 and 80 patients (9.9%) had a propensity score > 0.95. In the matched analyses, 35 of the 171 misdiagnosed patients died within 14 days (20.5%), versus 10 of the 171 correctly diagnosed patients (5.8%), yielding a difference of 14.6% (7.6%; 21.6%). In the total group of patients, the observed percentage of patients with an incorrect initial diagnosis that died within 14 days was 19.8% while propensity score reweighting estimated that their probability of dying would have been 6.5%, if they had been correctly diagnosed (difference 13.3% (95% CI 6.9%;19.6%)). After adjustment for all variables that showed disbalance in the propensity score a difference of 13.7% (7.4%; 19.9%) was estimated. Sensitivity analyses yielded similar results. However, performing weighted analyses without truncation yielded unstable results.

**Conclusion:**

Thus, we observed a substantial increase of 14 day mortality in initially misdiagnosed patients. Furthermore, several patients received propensity scores extremely close to one and were almost sure to be initially misdiagnosed.

**Supplementary Information:**

The online version contains supplementary material available at 10.1186/s12879-024-09299-9.

## Background

The diagnostic process is a key element of medicine but it is a complex process that is prone to errors. According to ‘Improving diagnosis in health care’ most people will experience misdiagnosis at least once in their life [1]. A previous study reported that the rate of disease-specific diagnostic errors ranged between 2.2 and 62.1% [2]. Infectious diseases are one of the three categories of diseases in which diagnostic errors can be most harmful to patients [3]. According to Abe et al. initial misdiagnosis of patients with infectious diseases was associated with an increase of in-hospital mortality of more than 10% [4].

However, investigating the relationship between initial misdiagnosis and mortality is challenging, as this relation can only be studied in observational data, where there is a high risk of confounding. The gold standard for investigating the effect of a treatment, or in this case a specific exposure, is a randomized controlled trial (RCT) [5]. For some interventions however- such as incorrect or correct diagnosis- it is impossible to conduct a RCT. Since it would be unethical to randomize the exposure ‘incorrect initial diagnosis’, a comparative observational study design is the only option. Propensity score methods, which have become increasingly popular over the past 15 years, can be applied to account for confounding [6–8]. However, these methods should be applied and reported carefully. In particular, the underlying assumptions of the propensity score methods should be likely to hold [9–13].

The aim of this study was to investigate the influence of initial misdiagnosis of the source of infection on 14 day mortality in a cohort of patients with bloodstream infection, using propensity score methods to address confounding.

## Methods

### Cohort description

Data from a previously described longitudinal cohort of patients diagnosed with monobacterial bloodstream infection (BSI) at the Leiden University Medical Centre (LUMC) between 2013 and 2015 were used [14]. Eligibility criteria of the current study were an episode of monobacterial BSI and an age of at least 18 years. Patients were excluded if all blood cultures were contaminated. All blood cultures with coagulase-negative staphylococci (CoNS) were classified as contaminated, because the probability of these cultures being contaminated is high [15, 16]. For other bacteria, the differentiation between contamination or true bacteraemia was made by the attending infectious diseases consultancy team at the time the blood culture results were reported. The infectious diseases consultancy team consisted of infectious diseases specialists and medical microbiologists. Standard empiric treatment for bacteraemia with unknown source of infection in the study centre was a second-generation cephalosporin, combined with gentamicin [14, 17].

### Data collection

Clinical and demographic data were retrieved from electronic patient files [17]. Clinical data consisted of medical history, source of infection, treatment received for bloodstream infection, clinical parameters such as fever and blood pressure, and scores indicating severity of illness. Severity of illness was expressed in PITT bacteraemia score (PBS) and the quick sequential organ failure assessment (qSOFA) score [18, 19]. Data from blood cultures were retrieved from the database of the Department of Medical Microbiology of the LUMC.

### Definition of exposure and outcomes

The exposure of interest was initial misdiagnosis. A patient was considered initially misdiagnosed if the suspected source of infection when a patient presented with fever did not match the final diagnosis of the source of infection or if the source of infection remained unidentified. The final diagnosis was made by the attending medical team after performing diagnostic tests such as blood sampling for cultures and imaging. Follow-up started at the day of blood sampling that resulted in a positive blood culture [17]. The outcome was 14 day mortality. Given that bloodstream infection is an acute condition it was expected that mortality related to initial misdiagnosis was most likely to occur within 2 weeks after diagnosis of bloodstream infection. A more long-term endpoint such as 30 day mortality would be more difficult to interpret because of competing causes of death [20, 21]. A short-term endpoint such as 7 day mortality would be too short to expect an effect of initial misdiagnosis on mortality.

### Confounders

Potential confounders to include in the propensity score model were selected based on prior clinical knowledge [22, 23]. Therefore all variables that were present at baseline and that were thought to be related to the exposure (initial misdiagnosis) and the outcome (14 day mortality) or the outcome only were identified [24, 25]. When variables were very similar (for example temperature and fever) we used the variable which was clinically most relevant, to prevent multicollinearity issues. The variables blood sampling from indwelling line and qSOFA score had a substantial number of missing values. For blood sampling from indwelling line it was assumed that a missing value indicated that a patient did not have an indwelling line. In case of qSOFA, missingness was handled by adding an indicator variable for missingness to the propensity model, as the reasons for missingness were less obvious. Thereafter complete case analysis was used which will yield valid treatment effects when treatment heterogeneity is absent [26].

### Baseline comparisons of exposure groups

Categorical variables were reported as numbers and percentages and continuous variables as means with standard deviations (SD) or medians with interquartile range (IQR), in case of skewed distributions. Demographic and clinical variables were compared between both exposure groups by calculating standardized mean differences (SMD).

### Propensity score analysis

In propensity score analysis, the target of estimation (the estimand) should be clearly defined, as this determines how propensity score methods should be conducted [27]. In this study we aimed to estimate the absolute decrease in 14 day mortality risk if all patients currently being initially misdiagnosed would have been correctly diagnosed. This is often called the average treatment effect on the treated (ATT). This is a risk difference and can be expressed as a difference in percentage points. For the purpose of this study we refer to it as the average effect of initial misdiagnosis on the misdiagnosed (AEMM). We estimated the AEMM by matching each patient being initially misdiagnosed to a patient with correct diagnosis using propensity score matching and by inverse probability weighting using propensity scores. Logistic regression models, with incorrect initial diagnosis (yes/no) as dependent variable were used to estimate propensity scores. The variables identified as potential confounders were entered in the propensity score model. Using the estimated propensity score, patients who were initially misdiagnosed were matched to patients with a correct initial diagnosis using nearest neighbour matching with a caliper of 0.02 and ratio of 1:1 without replacement. Balance between the matched groups was checked by calculating standardized mean differences (SMD) of variables included in the propensity score model. A variable was considered to be well balanced between the groups if the SMD < 0.10.

The distributions of the propensity scores in both exposure groups were visualized to evaluate whether the positivity assumption was violated.

In both matched subgroups, the percentage of patients who died within 14 days was calculated, together with the difference between the two percentages. The 95% confidence interval around the difference was calculated using robust standard errors.

Inverse probability of treatment weighting (IPTW) was performed as an alternative analysis. The same propensity score model was used. Weights were adjusted to estimate an AEMM (or ATT). Weights were truncated at the 99th percentile, to prevent extremely large weights, which may be very influential in the analysis. The average treatment effect of initial misdiagnosis on the misdiagnosed was calculated in the weighted cohort. The analysis was repeated with adjustment for all variables which showed some disbalance (SMD ≥ 0.10) in the propensity score [28]. Confidence intervals were calculated using robust standard errors.

STATA16.1 was used for analyses. The PSmatch package was used to perform propensity score matching in STATA16.1.

### Dealing with propensity scores close to 0 or 1 (non-positivity)

Additional sensitivity analyses were performed to gain insight in the influence of extreme propensity scores on the propensity score matching and weighting analyses. The additional analyses consisted of repeating the matching analysis varying the caliper and repeating the weighted analysis varying the cut-off values for truncation. Furthermore, clinical and demographic characteristics were compared between patients with propensity scores ≥0.95 and patients with propensity scores < 0.95 to explore which characteristics may be associated with an extremely high probability to get initially misdiagnosed.

### Unmeasured confounding

As propensity score methods rely on the ‘no unmeasured confounding’ assumption, the e-value was estimated for the point estimate of the primary propensity score matching analysis and for the lower limit of the corresponding confidence interval. The e-value represents the minimum strength of association of an unmeasured confounder with both the exposure as well as the outcome on the risk ratio scale to fully explain the observed effect of the exposure. The e-value was estimated using the e-value calculator provided by Mathur et al. and Van der Weele et al. [29, 30].

The STrengthening the Reporting of Observational studies in Epidemiology (STROBE) guidelines were followed for reporting results [31].

## Results

The database consisted of 893 patients with BSI. Six patients were excluded as data on whether or not first diagnosis was correct was missing. Thus, data of 887 patients were included in this study. A total of 341 patients (38.4%) received an incorrect first diagnosis. In total 95 (10.7%) patients died within 14 days; 65 (19.1%) of the patients who were initially misdiagnosed and 30 (5.5%) in the group of correctly diagnosed patients died within 14 days. Table 1 shows the demographic and clinical characteristics of the cohort. In Table 1 the variable high risk pathogen is included. In supplementary material S1 it is specified which bacteria were considered pathogens with a high risk of unfavourable clinical outcome [17]. Supplementary material S1 also includes a list of the causative pathogens that were included in the database.

**Table 1:** Clinical and demographic characteristics at baseline

	First diagnosis incorrect (***N*** = 341)	First diagnosis correct (***N*** = 546)	Number of missing values^**1**^	Standard mean difference (SMD)
	Median (IQR) or ***N*** (%)	Median (IQR) or ***N*** (%)	***N*** (%)	
Age	64 (54;72)	66 (54;73)		0.06
Male	213 (62.5)	317 (58.1)		−0.09
Blood sampling from an indwelling line	56 (16.4)	12 (2.2)	185 (20.9)	−0.57
Infection caused by anaerobic bacteria	17 (5.0)	19 (3.5)		−0.07
High risk pathogen	209 (61.3)	233 (42.7)		−0.38
Infection caused by gram-positive bacteria	187 (54.8)	195 (35.7)		−0.39
Presentation at outpatient clinic before hospital admission	137 (40.2)	362 (66.3)		0.54
Length of hospital stay ≥48 hours before developing bloodstream infection	162 (47.5)	122 (22.3)		−0.55
Pre-treatment with antibiotics before developing bloodstream infection	135 (39.8)	127 (23.3)	3 (0.3)	−0.36
Pre-treatment with antibiotic adequate	33 (9.7)	45 (8.2)	5 (0.6)	−0.05
Source of infection:				
• Urinary tract	42 (12.3)	189 (34.6)		0.54
• Gastro-intestinal	98 (28.7)	146 (26.7)		−0.04
• Pulmonary	17 (5.0)	72 (13.2)		0.29
• Intravascular	66 (19.4)	44 (8.1)		−0.33
• Skin and soft tissue	18 (5.3)	51 (9.3)		0.16
• Other	15 (4.4)	41 (7.5)		0.13
• Unknown	82 (24.0)	2 (0.4)		− 0.77
High risk source of infection	156 (45.8)	304 (55.7)		0.20
Antibiotic treatment in 2 months before developing bloodstream infection	194 (58.3)	238 (45.1)	26 (2.9)	−0.27
History of antibiotic resistance	57 (16.7)	73 (13.4)	19 (2.1)	−0.09
Infection caused by resistant gram-negative bacteria in past 6 months	21 (6.2)	35 (6.4)		0.01
ICU admission in past 6 months	47 (13.8)	35 (6.4)		−0.25
Admission to Dutch hospital in past 6 months	188 (55.1)	246 (45.1)		−0.20
Chronic urological disease	23 (6.8)	107 (19.9)	13 (1.5)	0.39
Lives in nursery home	14 (4.3)	16 (3.1)	38 (4.3)	−0.06
Immunocompromised	209 (61.3)	262 (48.0)		−0.27
Prednisolone use	131 (38.4)	142 (26.0)		−0.27
Diabetes mellitus	60 (17.6)	125 (22.9)		0.13
Neutropenia < 0.5 before developing bloodstream infection	87 (25.5)	26 (4.8)		−0.60
History of stem cell transplantation	46 (13.5)	24 (4.4)		−0.32
History of solid organ transplantation	33 (9.7)	82 (15.0)		0.16
Liver cirrhosis	8 (2.3)	12 (2.2)		−0.01
Current malignancy	129 (37.8)	133 (24.4)		−0.29
Dialysis	10 (2.9)	10 (1.8)	2 (0.2)	−0.07
Fever	187 (56.7)	346 (64.7)	22 (2.5)	0.16
Hypotension	57 (16.7)	99 (18.1)		0.04
Tachycardia	170 (49.9)	303 (55.5)		0.11
Altered state of consciousness (includes somnolence and confusion)	56 (16.4)	83 (15.2)		−0.03
Patient considered ‘ill’ by physician	91 (26.7)	130 (23.8)		−0.07
PITT bacteraemia score	1 (0;2)	1 (0;2)		−0.18
qSOFA score	1 (0;2)	1 (0;2)	84 (9.5)	0.12
qSOFA score missing	31 (9.1)	53 (9.7)		0.02
Resistance current pathogen	58 (17.0)	103 (18.9)		0.05
History of resistance of current pathogen and history of resistance known at baseline	21 (6.2)	30 (5.5)	1 (0.1)	−0.03
ICU admission for two days or more at time of blood sampling	31 (9.01)	19 (3.5)	1 (0.1)	−0.23

*ICU* Intensive care unit, *PITT score* PITT bacteremia score, *qSOFA score* Quick sequential organ failure assessment score, *SMD* Standardized mean difference

More details on the variables included in the propensity score model are presented in supplement S3

^1^When nothing is reported there were no missing values

### Building the propensity score model

The final propensity score model included the following variables: age in years, sex, blood sampling from indwelling line, infection caused by gram-positive bacteria, infection caused by anaerobic bacteria, infection caused by a high risk pathogen, presentation at outpatient clinic before hospital admission, length of hospital stay ≥48 hours before developing bloodstream infection (i.e. hospital acquired infection), intensive care unit (ICU) admission for 2 days or more at time of blood sampling (i.e. ICU acquired infection), pre-treatment with antibiotics before developing bloodstream infection, pre-treatment adequate, source of infection: urinary tract (yes/no), source of infection: gastro-intestinal, source of infection: pulmonary, source of infection: intravascular, source of infection: skin and soft tissue, other source of infection, source of infection with high risk of unfavourable clinical outcome, antibiotic treatment in 2 months before developing bloodstream infection, history of antibiotic resistance, infection by resistant gram-negative bacteria in past 6 months, ICU admission in past 6 months, admission to Dutch hospital in past 6 months, chronic urological disease, lives in nursery home, immunocompromised, prednisolone use, diabetes mellitus, neutropenia, history of stem cell transplantation, history of solid organ transplantation, liver cirrhosis, current malignancy, dialysis, fever, hypotension, tachycardia, altered state of consciousness, PITT bacteraemia score, qSOFA score, qSOFA missing value (yes/no), history of resistance of current pathogen and history of resistance known at baseline, resistance current pathogen and patient considered ‘ill’ by physician.

All identified potential confounders and the subset of variables used to prevent multicollinearity can be found in supplementary material S2A and S2B. The variable hospital admission abroad before admission for bloodstream infection was excluded from the propensity score model because only five patients had been admitted abroad and this variable predicted incorrect diagnosis perfectly. Additional explanatory notes on the variables included in the propensity score model are presented in the supplementary material S3.

**Fig. 1:**
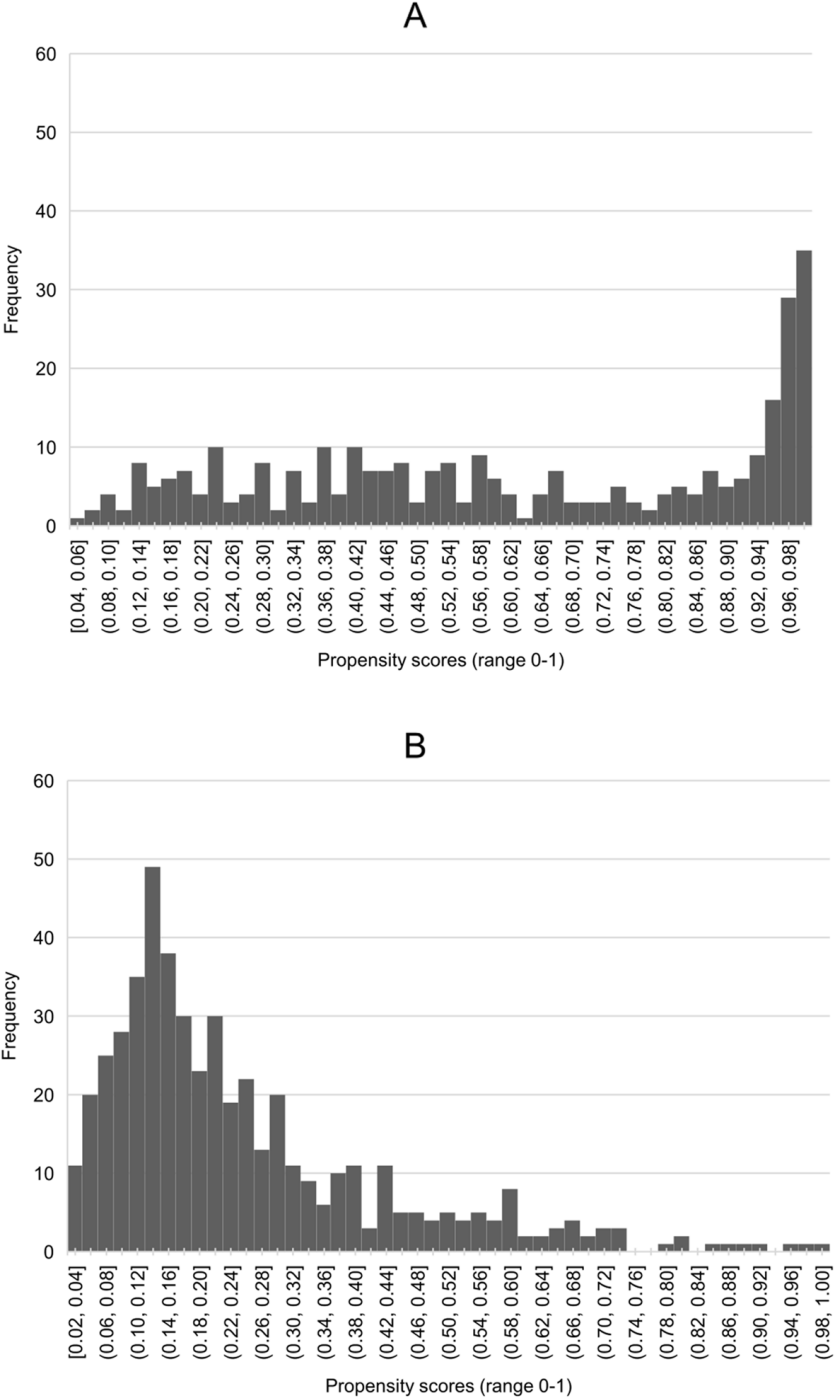
Distribution of propensity scores for incorrect and correct initial diagnosis 1A. Distribution of propensity scores in patients who received incorrect initial diagnosis 1B. Distribution of propensity scores in patients who received correct initial diagnosis

For 806 patients (90.9%) the final propensity score was calculated, 81 patients with missing values in one of the variables of the model were excluded from further analyses. In supplementary material S2B missing data of all variables that were used to build the propensity score model are shown. Propensity scores ranged between 0.015 and 0.999 (Fig. 1). In total 27 patients (3.3%) had a propensity score below 0.05 and 80 patients (9.9%) had a propensity score > 0.95. Figure 1 shows that there was some degree of overlap in propensity scores between treatment groups.

### Propensity score matching

**Fig. 2:**
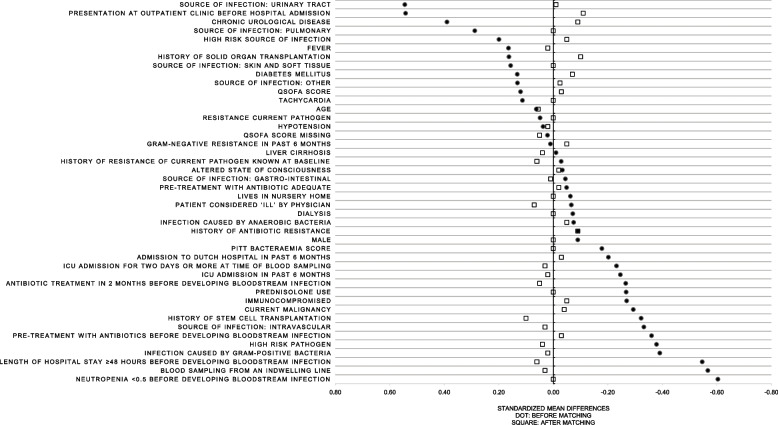
Standardized mean differences before and after matching. QSOFA score: quick sequential organ failure assessment score

Of 806 patients 171 misdiagnosed patients were matched to 171 correctly diagnosed patients. Propensity scores in the matched cohort ranged from 0.043 to 0.986. The distribution of baseline characteristics after matching is shown in Table 2. Nearly all standardized mean differences of variables included in the propensity score model were < 0.10 after matching. In Fig. 2 the standardized mean differences before and after matching are depicted. After matching some disbalance (SMD ≥ 0.10) was observed for presentation at outpatient clinic (SMD = 0.11), history of stem cell transplantation (0.10) and solid organ transplantation (0.10).

**Table 2:** Clinical and demographic characteristics after matching

	First diagnosis incorrect (***N*** = 171)	First diagnosis correct (***N*** = 171)	Standard mean difference (SMD)
	Median (IQR) or ***N*** (%)	Median (IQR) or ***N*** (%)	
Age	65 (54;74)	67 (54;75)	0.06
Male	107 (62.6)	107 (62.6)	0.00
Blood sampling from an indwelling line	8 (4.7)	9 (5.3)	0.03
Infection caused by anaerobic bacteria	11 (6.4)	9 (5.3)	−0.05
High risk pathogen	89 (52.1)	92 (53.8)	0.04
Infection caused by gram-positive bacteria	78 (45.6)	80 (46.8)	0.02
Presentation at outpatient clinic before hospital admission	92 (53.8)	83 (48.5)	− 0.11
Length of hospital stay ≥48 hours before developing bloodstream infection	56 (32.8)	61 (35.7)	0.06
Pre-treatment with antibiotics before developing bloodstream infection	50 (29.3)	48 (28.1)	−0.03
Pre-treatment with antibiotic adequate	15 (8.8)	14 (8.2)	−0.02
Source of infection:			
• Urinary tract	36 (21.1)	35 (20.5)	−0.01
• Gastro-intestinal	59 (34.5)	60 (35.1)	0.01
• Pulmonary	14 (8.2)	14 (8.2)	0.00
• Intravascular	34 (19.9)	32 (18.7)	0.03
• Skin and soft tissue	14 (8.2)	14 (8.2)	0.00
• Other	12 (7.0)	11 (6.4)	−0.02
• Unknown	4 (2.3)	2 (1.2)	−0.09
High risk source of infection	66 (38.6)	62 (36.3)	−0.05
Antibiotic treatment in 2 months before developing bloodstream infection	84 (49.1)	88 (51.5)	0.05
History of antibiotic resistance	21 (12.3)	16 (9.4)	−0.09
Infection caused by resistant gram-negative bacteria in past 6 months	10 (5.9)	8 (4.7)	−0.05
ICU admission in past 6 months	16 (9.4)	17 (9.9)	0.02
Admission to Dutch hospital in past 6 months	86 (50.3)	83 (48.5)	−0.03
Chronic urological disease	15 (8.8)	11 (6.4)	−0.09
Lives in nursery home	6 (3.5)	6 (3.5)	0.00
Immunocompromised	88 (51.5)	84 (49.1)	−0.05
Prednisolone use	51 (29.8)	51 (29.8)	0.00
Diabetes mellitus	35 (20.5)	30 (17.5)	−0.07
Neutropenia < 0.5 before developing bloodstream infection	22 (12.9)	22 (12.9)	0.00
History of stem cell transplantation	7 (4.1)	11 (6.4)	0.10
History of solid organ transplantation	20 (11.7)	15 (8.8)	−0.10
Liver cirrhosis	4 (2.3)	5 (2.9)	0.04
Current malignancy	59 (34.5)	56 (32.8)	−0.04
Dialysis	5 (2.9)	5 (2.9)	0.00
Fever	99 (57.9)	101 (59.1)	0.02
Hypotension	25 (14.6)	26 (15.2)	0.02
Tachycardia	89 (52.1)	89 (52.1)	0.00
Altered state of consciousness (includes somnolence and confusion)	24 (14.0)	23 (13.5)	−0.02
Patient considered ‘ill’ by physician	43 (25.2)	48 (28.1)	0.07
PITT bacteraemia score	1 (0;2)	1 (0;2)	0.00
qSOFA score	1 (0;2)	1 (0;1)	−0.03
qSOFA score missing	8 (4.7)	10 (5.9)	0.05
Resistance current pathogen	30 (17.5)	30 (17.5)	0.00
History of resistance of current pathogen and history of resistance known at baseline	7 (4.1)	9 (5.3)	0.06
ICU admission for two days or more at time of blood sampling	6 (3.5)	7 (4.1)	0.03

*ICU* Intensive care unit, *PITT score* PITT bacteremia score, *qSOFA score* Quick sequential organ failure assessment score, *SMD* Standardized mean difference

More details on the variables included in the propensity score model are presented in supplement S3

In Table 3 results of all matching and IPTW analyses are presented. Of the 171 misdiagnosed matched patients, 35 patients died (20.5%) while of the 171 correctly diagnosed patients 10 patients died (5.8%). This yielded an estimated effect of initial misdiagnosis on mortality of 14.6% (7.6%;21.6%).

**Table 3:** Propensity score analysis of influence of initial misdiagnosis on 14 day mortality

Type of analysis	AEMM% (95%CI)	AEMM% (95%CI) Double adjusted^**2**^	N matched pairs
Matching, caliper 0.02	14.6 (7.6;21.6)	Not applicable	171
*Additional*^*1*^: Matching, caliper 0.2	13.5 (6.5;20.4)	Not applicable	171
*Additional*: Matching, caliper 0.5	13.5 (7.0;20.0)	Not applicable	192
IPTW truncated (weights >99th percentile)	13.3 (6.9;19.6)	13.7 (7.4;19.9)	Not applicable
*Additional*: IPTW truncated (weights > 10)	11.8 (3.0;20.6)	12.3 (4.1;20.6)	Not applicable
*Additional*: IPTW untruncated	−4.2 (−36.1;27.7)	− 0.1 (−23.8;23.5)	Not applicable

*AEMM* Average effect of initial misdiagnosis on the misdiagnosed, *CI* Confidence interval, *IPTW* Inversed probability of treatment weighting

^1^Additional analyses were performed after primary analyses to gain insight in the influence of extreme propensity scores on results

^2^Results of IPTW adjusted for presentation at outpatient clinic before hospital admission, history of stem cell transplantation and history of solid organ transplantation in final outcome analysis

### Propensity score weighting

Propensity score weights for the AEMM analysis ranged between 0.016 and 71. Three weights were larger than 10 respectively 15, 26 and 71, which were truncated to 4.2 (cut-off value for truncation at 99th percentile).

The observed percentage of patients with an initial misdiagnosis that died within 14 days was 19.8% while their estimated probability of dying would have been 6.5%, if they had been correctly diagnosed. This yielded an AEMM of 13.3% (6.9%; 19.6%). After adjustment for presentation at outpatient clinic before hospital admission, history of stem cell transplantation and history of solid organ transplantation an AEMM of 13.7% (7.4%; 19.9%) was estimated.

### Propensity scores close to 0 or 1 (non-positivity)

As propensity scores close to 1 were observed, the matching analysis was repeated varying the caliper (Table 3) in the matched analyses. When caliper was widened to 0.2 a total of 171 initially misdiagnosed patients were matched to 171 correctly diagnosed patients. An AEMM of 13.5% (6.5%; 20.4%) was estimated. Matched analysis with caliper of 0.5 yielded 192 matched pairs and an AEMM of 13.5% (7.0%; 20.0%).

The IPTW analysis was repeated using varying truncation cut-off points. When the cut-off value for truncation was set on the absolute value of 10 the AEMM was 11.8% (3.0%; 20.6%) and 12.3% (4.1%; 20.6%) after double adjustment. Using the original weights, (not truncated) yielded unstable results with large 95% confidence intervals, AEMM: − 4.2% (− 36.1%; 27.7%).

Table 4 shows the distribution of clinical and demographic characteristics in patients with extremely high propensity scores (≥0.95) and patients with propensity scores < 0.95. Patients with propensity scores close to one were relatively frequently infected by a high risk pathogen and were more frequently infected by a gram-positive pathogen. They also had more frequently hospital acquired infection, had more frequently ICU acquired infection, had more often been admitted to an ICU in the past 6 months, had more frequently a malignancy, were more frequently immunocompromised or neutropenic, had more often a history of stem cell transplantation and were more frequently prednisolone users. Furthermore, in patients with extremely high propensity scores the source of infection remained unclear in most (87.5%) patients. Patients with propensity scores < 0.95 had more often a history of chronic urological disease and were more frequently admitted to the hospital from an outpatient clinic.

**Table 4:** Clinical and demographic characteristics of patients with propensity scores of ≥0.95 versus < 0.95

	Propensity score 0.95 or higher (***N*** = 80)	Propensity score < 0.95 (***N*** = 726)	Standard mean difference (SMD)
	Median (IQR) or ***N*** (%)	Median (IQR) or ***N*** (%)	
Age	66 (56;74)	66 (54;73)	0.00
Male	55 (68.8)	427 (58.8)	−0.21
Blood sampling from an indwelling line	21 (26.3)	43 (5.9)	−0.57
Infection caused by anaerobic bacteria	2 (2.5)	32 (4.4)	0.10
High risk pathogen	59 (73.8)	340 (46.8)	−0.57
Infection caused by gram-positive bacteria	51 (63.8)	291 (40.1)	−0.49
Presentation at outpatient clinic before hospital admission	24 (30.0)	438 (60.3)	0.64
Length of hospital stay ≥48 hours before developing bloodstream infection	47 (58.8)	202 (27.8)	−0.65
Pre-treatment with antibiotics before developing bloodstream infection	35 (43.8)	198 (27.3)	−0.35
Pre-treatment with antibiotic adequate	8 (10.0)	61 (8.4)	−0.06
Source of infection:			
• Urinary tract	0	214 (29.5)	0.91
• Gastro-intestinal	3 (3.8)	224 (30.9)	0.77
• Pulmonary	0	81 (11.2)	0.50
• Intravascular	7 (8.8)	89 (12.3)	0.11
• Skin and soft tissue	0	61 (8.4)	0.43
• Other	0	44 (6.1)	0.36
• Unknown	70 (87.5)	9 (1.2)	−3.48
High risk source of infection	70 (87.5)	348 (47.9)	−0.93
Antibiotic treatment in 2 months before developing bloodstream infection	46 (57.5)	350 (48.2)	−0.19
History of antibiotic resistance	17 (21.3)	101 (13.9)	−0.19
Infection caused by resistant gram-negative bacteria in past 6 months	7 (8.8)	45 (6.2)	−0.10
ICU admission in past 6 months	18 (22.5)	54 (7.4)	−0.43
Admission to Dutch hospital in past 6 months	45 (56.3)	346 (47.7)	−0.17
Chronic urological disease	1 (1.3)	119 (16.4)	0.55
Lives in nursery home	4 (5.0)	25 (3.4)	−0.08
Immunocompromised	52 (65.0)	368 (50.7)	−0.29
Prednisolone use	36 (45.0)	202 (27.8)	−0.36
Diabetes mellitus	12 (15.0)	150 (20.7)	0.15
Neutropenia < 0.5 before developing bloodstream infection	29 (36.3)	75 (10.3)	−0.64
History of stem cell transplantation	13 (16.3)	45 (6.2)	−0.32
History of solid organ transplantation	7 (8.8)	99 (13.5)	0.15
Liver cirrhosis	2 (2.5)	15 (2.1)	−0.03
Current malignancy	36 (45.0)	206 (28.4)	−0.35
Dialysis	2 (2.5)	13 (1.8)	−0.05
Fever	41 (51.3)	455 (62.7)	0.23
Hypotension	17 (21.3)	125 (17.2)	−0.10
Tachycardia	35 (43.8)	382 (52.6)	0.18
Altered state of consciousness (includes somnolence and confusion)	19 (23.8)	107 (14.7)	−0.23
Patient considered ‘ill’ by physician	19 (23.8)	169 (23.3)	−0.01
PITT bacteraemia score	1 (0;2)	1 (0;2)	−0.35
qSOFA score	1 (0;2)	1 (0;2)	−0.04
qSOFA score missing	8 (10.0)	55 (7.6)	−0.09
Resistance current pathogen	11 (13.8)	136 (18.7)	0.13
History of resistance of current pathogen and history of resistance known at baseline	9 (11.3)	37 (5.1)	−0.23
ICU admission for two days or more at time of blood sampling	10 (12.5)	27 (3.7)	−0.32

*ICU* Intensive care unit, *PITT score* PITT bacteremia score, *qSOFA score* Quick sequential organ failure assessment score, *SMD* Standardized mean difference

More details on the variables included in the propensity score model are presented in supplement S3

### Unmeasured confounding

An e-value of 6.46 was estimated for the point estimate of the primary propensity score matching analysis and an e-value of 3.29 was estimated for the under bound of the corresponding 95% confidence interval.

## Discussion

In this study the influence of initial misdiagnosis in patients who developed bloodstream infection on 14 day mortality was investigated using propensity score matching and weighting. We estimated that in patients who were initially misdiagnosed 14 day mortality was substantially higher (difference 14.6% matching and 13.7% weighting) than what the mortality risk would have been if these patients had been correctly diagnosed. Sensitivity analyses consisting of changing caliper and truncation of the weights did not substantially change the results. Performing weighted analyses without truncation yielded very unstable results.

### Impact of misdiagnosis on mortality

In previous studies, infections, vascular events and cancers have been identified as the big three diseases that account for the majority of mortality and morbidity due to misdiagnosis [2]. In 2019 Abe et al. published an article on the effect of initial misdiagnosis of site of infection in patients with infection on in-hospital mortality [4]. Initial misdiagnosis occurred in 11.6% of patients, versus 38.4% in our population. It was reported that mortality was increased with > 10% in patients who were misdiagnosed at admission, which is a similar result to what was observed in this study. It would be rational to assume that the increased 14 day mortality in the group of misdiagnosed patients was due to delayed adequate antibiotic treatment. In several previous studies, it was observed that delayed adequate antibiotic treatment was associated with increased mortality [32, 33]. However, the association between delayed adequate antibiotic treatment and mortality can only be investigated in observational studies as it would be unethical to conduct a randomized controlled trial. Therefore, studies in which the effect of delayed appropriate antibiotic treatment on mortality is investigated are at risk for confounding. In 2020 Lambregts et al. investigated the effect of initial inadequate empirical treatment on 14 day mortality in the BSI cohort that was also used in this study, using propensity score matching and IPTW to adjust for confounding [17]. It was estimated that delayed adequate antibiotic treatment did not statistically significantly influence 14 day mortality. Thus, it seems that the influence of initial misdiagnosis on mortality can at least not entirely be explained by a delay of appropriate antibiotic treatment. An alternative partial explanation for increased mortality in initially misdiagnosed patients could be inadequate source control. For example, Tellor et al. showed that in patients with intra-abdominal sepsis and associated bacteraemia inadequate source control was a determinant for mortality, independent from inadequate antibiotic therapy [34].

### Issues encountered in propensity score matching and propensity score weighting

In this study several patients received propensity scores extremely close to one, meaning that there was a group of patients that had an extremely high probability to be misdiagnosed. We explored which clinical and demographic variables were associated with extremely high propensity scores. In the group of patients with propensity scores extremely close to one we observed, among other things, more patients who had a malignancy, more patients who were immunocompromised, more recent ICU admissions, more hospital acquired bacteraemia and more infections caused by high risk pathogens. Moreover, most of the patients with extremely high propensity scores never received a correct diagnosis. In order to be able to identify high risk patients at admission in the future, it is important to take into account clinical and demographic characteristics that are associated with an extremely high probability to be initially misdiagnosed.

Furthermore, propensity scores close to one give rise to methodological issues. Patients with extreme propensity values are difficult to match. One could decide to  make close matches (small caliper), leading to discarding a large part of the observations, including some of the mis-diagnosed patients with very high propensity values. In that case, a different effect is estimated: the average effect of initial misdiagnosis on the misdiagnosed which were matchable. Using less strict matches, may also result in biased estimate of the AEMM due to residual confounding. For inverse probability weighing, very high weights may occur. By varying truncation cut-off points in IPTW analyses we illustrated that including extremely large propensity scores weights yielded unstable results with a large variance. Using truncated weights reduced the variance substantial.

### Strengths and limitations

A strength of this study is that propensity score matching and weighting were applied to adjust for confounding, which allowed us to balance possible confounders between the two exposure groups. Another strength of the study was that because two different propensity score methods were used and additional analyses were performed after propensity score matching and weighting, we gained better understanding of the influence of both propensity score methods on the results. Therefore we were able to draw conclusions more carefully.

A limitation of the study is that the analysed data were derived from a cohort study that was conducted between 2013 and 2015 because more recent data were not available. However, the standard empiric treatment of bloodstream infection of unknown origin between 2013 and 2015 consisted of a second-generation cephalosporin combined with gentamicin, which still is the standard empiric treatment in the study centre. Another limitation of this study is that 81 patients (9.1%) of the initial cohort were excluded from analyses due to missing values in variables included in the propensity score model. However, this percentage is relatively small and in none of the variables in the final propensity score model the percentage of missing values was larger than 5% except for qSOFA score. Furthermore, because several patients received a propensity score close to one, a certain degree of non-positivity existed. For propensity score matching this meant that many patients could not be matched. In this study 50.1% of patients who received incorrect first diagnosis were matched. In IPTW analyses all patients for whom a propensity score was calculated (90.9%) were used in analysis. Another limitation is that propensity score methods are based on the underlying assumption that there is no unmeasured confounding. It is impossible to be sure that there are no unmeasured confounders. However, the estimated e-value for the point estimate and for the under bound of the 95% CI for the matching analysis were large, meaning that unmeasured confounding had to be substantial to explain the increased mortality after initial misdiagnosis [30]. Furthermore, we are quite convinced that the most relevant possible confounders were included in the propensity score model, because the list of possible confounders was extensive and consisted of 94 variables.

## Conclusions

From this propensity score matched and weighted study it can be concluded that an incorrect first diagnosis of site of infection in patients with BSI increased 14 day mortality with 14.6%. Initial misdiagnosis can therefore be seen as a marker of poor prognosis. Moreover, several patients received a propensity score extremely close to one, meaning that several patients were almost sure to be misdiagnosed. These results stress the importance of a correct initial diagnosis of the site of infection despite of the use of broad spectrum antibiotics as empirical therapy for BSI. To mitigate future mortality, it is imperative to develop strategies aimed at expediting and enhancing the diagnostic process, particularly in cases where the source of infection remains unknown. One potential intervention involves convening a multi-disciplinary consultation within 24 hours of hospital admission when the source of infection persists unclear, or adopting a lower threshold for diagnostic imaging, especially for patients with a markedly high likelihood of initial misdiagnosis. To avert misdiagnoses and consequent mortality in the future, a deeper understanding of patients with BSI that at baseline have an extremely high probability to be misdiagnosed is essential.

### Supplementary Information

**Supplementary Material 1.**

## Data Availability

The data that were used for this study are available from the corresponding author upon reasonable request.

## Abbreviations

AEMM: Average effect of initial misdiagnosis on the misdiagnosed
ATT: Average treatment effect on the treated
BSI: Bloodstream infection
CoNS: Coagulase-negative staphylococci
ICU: Intensive care unit
IPTW: Inversed probability of treatment weighting
IQR: Interquartile range
PBS: PITT bacteraemia score
qSOFA: Quick sequential organ failure assessment
RCT: Randomized controlled trial
SD: Standard deviation
SMD: Standardized mean difference
Spp.: Species (plural)
