# Supplementary material for: Influence of initial misdiagnosis on mortality in patients with bacteraemia: propensity score matching and propensity score weighting analyses
Source: BMC Infect Dis. 2024 Apr 11;24:389. doi: 10.1186/s12879-024-09299-9 (PMC11007998; doi:10.1186/s12879-024-09299-9)
Supplement: Supplementary file 1 — Supplementary Material 1. [file 12879_2024_9299_MOESM1_ESM.docx]

BMC Infectious Diseases

Influence of initial misdiagnosis on mortality in patients with bacteraemia: propensity score matching and propensity score weighting analyses.

Anna M. Eikenboom, Merel M. C. Lambregts, Mark G.J. de Boer, Saskia Le Cessie

Supplementary Material

[Appendix S1 – Categories of causative pathogens included in the database 2](#_Toc158044899)

[Appendix S2 – Potential confounders 3](#_Toc158044900)

[S2 A. Variables that are considered possible confounders 3](#_Toc158044901)

[S2 B. Confounders to be included to build the propensity score model 6](#_Toc158044902)

[Appendix S3 – Explanatory notes on confounders included in the propensity score model 8](#_Toc158044903)

[Appendix S4 – STATA code that was used for analyses 10](#_Toc158044904)

# Appendix S1 – Categories of causative pathogens included in the database

Categories of pathogens:

Pathogens marked with an asterisk were considered high risk pathogens.

Streptococcus pneumoniae*

Streptococcus spp. other than streptococcus pneumoniae*

Enterococcus spp.*

Staphylococcus aureus*

Pseudomonas aeruginosa*

Escherichia coli

Klebsiella spp.

Enterobacter spp.

Serratia spp.

Proteus mirabilis

Other anaerobes (not specified in other categories)*

Other bacteria (not specified in other categories)*

# Appendix S2 – Potential confounders

## S2 A. Variables that are considered possible confounders

List A includes variables:

- That were present at baseline
- That were thought to be related to initial misdiagnosis and 14 day mortality or 14 day mortality alone
- That do not act as mediators on the causal pathway

| **Variables that are considered possible confounders** |
| --- |
| 1. Age |
| 1. Age >50 (years) |
| 1. Age >55 |
| 1. Age >60 |
| 1. Age >65 |
| 1. Age >70 |
| 1. Age >75 |
| 1. Sex |
| 1. Blood sampling from an indwelling line |
| 1. Streptococcus infection (s. pneumoniae included) |
| 1. Streptococcus infection (s. pneumoniae excluded) |
| 1. S. pneumoniae infection |
| 1. Enterococcus infection |
| 1. S. aureus infection |
| 1. E. coli infection |
| 1. Klebsiella species infection |
| 1. Pseudomonas infection |
| 1. Enterobacter infection |
| 1. Proteus mirabilis infection |
| 1. Serratia species infection |
| 1. Infection caused by anaerobic bacteria |
| 1. Infection caused by other bacteria (other than streptococcus, enterococcus, e. coli, klebsiella species, peudomonas, enterobacter, proteus mirabilis, serratia, anaerobe bacteria) |
| 1. Infection caused by bacteria that are considered pathogens with a high risk of unfavourable clinical outcome |
| 1. Infection caused by gram-positive bacteria |
| 1. Infection caused by gram-negative bacteria |
| 1. Presentation at outpatient clinic before hospital admission |
| 1. Length of hospital stay ≥48 hours before developing blood stream infection |
| 1. Pre-treatment with antibiotics before developing blood stream infection |
| 1. Pre-treatment with antibiotic adequate |
| 1. Source of infection: urinary tract |
| 1. Source of infection: gastro-intestinal |
| 1. Source of infection: pulmonary |
| 1. Source of infection: intravascular |
| 1. Source of infection: skin and soft tissue |
| 1. Source of infection: other source |
| 1. Source of infection with high risk of unfavourable clinical outcome (urinary tract, gastro-intestinal, pulmonary, other than specified source, unidentified) |
| 1. Antibiotic treatment in 2 months before developing blood stream infection |
| 1. Stay abroad before hospital admission |
| 1. History of antibiotic resistance |
| 1. Infection caused by resistant gram-negative bacteria in past 6 months |
| 1. Hospital admission abroad before admission for blood stream infection |
| 1. ICU admission in past 6 months |
| 1. Admission to Dutch hospital in past 6 months |
| 1. Chronic urological disease |
| 1. Lives in nursery home |
| 1. Immunocompromised |
| 1. Prednisolone use |
| 1. Diabetes mellitus |
| 1. Neutropenia <0.5 before developing blood stream infection |
| 1. History of stem cell transplantation |
| 1. History of solid organ transplantation |
| 1. Hematological malignancy |
| 1. Immunocompromised due to disease or treatment other than prednisolone use, neutropenia, history of transplantation, hematological malignancy |
| 1. Liver cirrhosis |
| 1. Solid malignancy |
| 1. Current malignancy |
| 1. Dialysis |
| 1. Temperature |
| 1. Fever |
| 1. Systolic blood pressure |
| 1. Hypotension |
| 1. Heart rate |
| 1. Tachycardia |
| 1. Respiratory rate |
| 1. Confused |
| 1. Somnolent |
| 1. Sedated |
| 1. Altered state of consciousness (includes somnolence and confusion) |
| 1. Arterial oxygen saturation |
| 1. Respiratory rate >22 per minute |
| 1. Oxygen support |
| 1. Patient considered ‘ill’ by physician |
| 1. Patient considered ‘moderately ill’ by physician |
| 1. Patient considered ‘not ill’ by physician |
| 1. Unknown whether patient is considered ill by physician |
| 1. PITT bacteraemia score – temperature |
| 1. PITT bacteraemia score – hypotension |
| 1. PITT bacteraemia score – hypotension with need of inotropic support |
| 1. PITT bacteraemia score – mechanical ventilation |
| 1. PITT bacteraemia scire – neurology (mental status) |
| 1. PITT bacteraemia score |
| 1. PITT bacteraemia score ≥2 |
| 1. qSOFA score |
| 1. Resistance current pathogen |
| 1. Presence of ESBL |
| 1. Presence HLG |
| 1. Presence of beta-lactamase |
| 1. Resistance against cefuroxime/gentamycin |
| 1. Resistance against cefuroxime |
| 1. Resistance against gentamycin |
| 1. Resistance against ciprofloxacin |
| 1. Infection by amoxicillin resistant enterococcus |
| 1. History of resistance of current pathogen and history of resistance known at baseline |
| 1. ICU admission for two days or more at time of blood sampling |

## S2 B. Confounders to be included to build the propensity score model

List B includes variables:

- That were present at baseline
- That were thought to be related to initial misdiagnosis and 14 day mortality or 14 day mortality alone
- That do not act as mediators on the causal pathway
- Variables that were considered clinically most relevant

| **Variables that are considered possible confounders** | **Number (%) of missing values** |
| --- | --- |
| 1. Age | 0 |
| 1. Sex | 0 |
| 1. Blood sampling from an indwelling line | 185 (20.9) |
| 1. Infection caused by anaerobic bacteria | 0 |
| 1. Infection caused by bacteria that are considered pathogens with a high risk of unfavourable clinical outcome | 0 |
| 1. Infection caused by gram-positive bacteria | 0 |
| 1. Presentation at outpatient clinic before hospital admission | 0 |
| 1. Length of hospital stay ≥48 hours before developing blood stream infection | 0 |
| 1. Pre-treatment with antibiotics before developing blood stream infection | 3 (0.3) |
| 1. Pre-treatment with antibiotic adequate | 5 (0.6) |
| 1. Source of infection: urinary tract | 0 |
| 1. Source of infection: gastro-intestinal | 0 |
| 1. Source of infection: pulmonary | 0 |
| 1. Source of infection: intravascular | 0 |
| 1. Source of infection: skin and soft tissue | 0 |
| 1. Source of infection: other source | 0 |
| 1. Source of infection with high risk of unfavourable clinical outcome (urinary tract, gastro-intestinal, pulmonary, other than specified source, unidentified) | 0 |
| 1. Antibiotic treatment in 2 months before developing blood stream infection | 26 (2.9) |
| 1. History of antibiotic resistance | 19 (2.1) |
| 1. Infection caused by resistant gram-negative bacteria in past 6 months | 0 |
| 1. Hospital admission abroad before admission for blood stream infection | 19 (2.1) |
| 1. ICU admission in past 6 months | 0 |
| 1. Admission to Dutch hospital in past 6 months | 0 |
| 1. Chronic urological disease | 13 (1.5) |
| 1. Lives in nursery home | 38 (4.3) |
| 1. Immunocompromised | 0 |
| 1. Prednisolone use | 0 |
| 1. Diabetes mellitus | 0 |
| 1. Neutropenia <0.5 before developing blood stream infection | 0 |
| 1. History of stem cell transplantation | 0 |
| 1. History of solid organ transplantation | 0 |
| 1. Liver cirrhosis | 0 |
| 1. Current malignancy | 0 |
| 1. Dialysis | 2 (0.2) |
| 1. Fever | 22 (2.5) |
| 1. Hypotension | 0 |
| 1. Tachycardia | 0 |
| 1. Altered state of consciousness (includes somnolence and confusion) | 0 |
| 1. Patient considered ‘ill’ by physician | 0 |
| 1. PITT bacteraemia score | 0 |
| 1. qSOFA score | 84 (9.5) |
| 1. Resistance current pathogen | 0 |
| 1. History of resistance of current pathogen and history of resistance known at baseline | 1 (0.1) |
| 1. ICU admission for two days or more at time of blood sampling | 1 (0.1) |

# Appendix S3 – Explanatory notes on confounders included in the propensity score model

| **Confounder** | **Explanatory notes** |
| --- | --- |
| 1. Age |  |
| 1. Sex |  |
| 1. Blood sampling from an indwelling line |  |
| 1. Infection caused by anaerobic bacteria | Although at baseline the pathogen that is causing the infection is unknown, the pathogen is present at baseline. Blood cultures that are sampled at baseline eventually show which pathogen caused the infection.^1^ |
| 1. Infection caused by bacteria that are considered pathogens with a high risk of unfavourable clinical outcome | See explanatory note^1^ |
| 1. Infection caused by gram-positive bacteria | See explanatory note^1^ |
| 1. Presentation at outpatient clinic before hospital admission |  |
| 1. Length of hospital stay ≥48 hours before developing blood stream infection |  |
| 1. Pre-treatment with antibiotics before developing blood stream infection |  |
| 1. Pre-treatment with antibiotic adequate |  |
| 1. Source of infection: urinary tract | The source of infection usually is discovered after medical examination, blood work, imaging and positive blood cultures. Although the source of infection is unknown at baseline, the infection is already present in the patient at a specific site, such as the urinary tract or skin.  The initial diagnosis of the source of infection that is made shortly after admission does not influence the original source of infection. Therefore, the source of infection does not act as a mediator on the causal pathway.^2^ |
| 1. Source of infection: gastro-intestinal | See explanatory note^2^ |
| 1. Source of infection: pulmonary | See explanatory note^2^ |
| 1. Source of infection: intravascular | See explanatory note^2^ |
| 1. Source of infection: skin and soft tissue | See explanatory note^2^ |
| 1. Source of infection: other source | See explanatory note^2^ |
| 1. Source of infection with high risk of unfavourable clinical outcome (urinary tract, gastro-intestinal, pulmonary, other than specified source, unidentified) | See explanatory note^2^ |
| 1. Antibiotic treatment in 2 months before developing blood stream infection |  |
| 1. History of antibiotic resistance |  |
| 1. Infection caused by resistant gram-negative bacteria in past 6 months |  |
| 1. ICU admission in past 6 months |  |
| 1. Admission to Dutch hospital in past 6 months |  |
| 1. Chronic urological disease |  |
| 1. Lives in nursery home |  |
| 1. Immunocompromised |  |
| 1. Prednisolone use |  |
| 1. Diabetes mellitus |  |
| 1. Neutropenia <0.5 before developing blood stream infection | Patients who were neutropenic before developing blood stream infection (absolute blood neutrophil count <0.5 x 10^9/L) |
| 1. History of stem cell transplantation |  |
| 1. History of solid organ transplantation |  |
| 1. Liver cirrhosis |  |
| 1. Current malignancy |  |
| 1. Dialysis |  |
| 1. Fever |  |
| 1. Hypotension |  |
| 1. Tachycardia |  |
| 1. Altered state of consciousness (includes somnolence and confusion) |  |
| 1. Patient considered ‘ill’ by physician |  |
| 1. PITT bacteraemia score | Clinical score that is assessed at baseline |
| 1. qSOFA score | Clinical score that is assessed at baseline |
| 1. qSOFA score missing | Missing indicator variable for qSOFA score |
| 1. Resistance current pathogen | See explanatory note ^1^ |
| 1. History of resistance of current pathogen and history of resistance known at baseline | See explanatory note ^1^ |
| 1. ICU admission for two days or more at time of blood sampling |  |

# Appendix S4 – STATA code that was used for analyses

* Explore database

codebook

* Unadjusted effect of misdiagnosis on 14 day mortality

tab1 FocusOnjuist_opname_koorts Mortaliteit14

tabstat Mortaliteit14 ,by(FocusOnjuist_opname_koorts) s(mean) nototal

* Install programs

ssc install psmatch2

ssc install stddiff

ssc install covbal

* Exclude if missing value in exposure

drop if FocusOnjuist_opname_koorts==.

* Table with missing values

mdesc

* Table 1 before matching

sort FocusOnjuist_opname_koorts

by FocusOnjuist_opname_koorts: tab Sexe

by FocusOnjuist_opname_koorts: tab afname_uit_lijn

by FocusOnjuist_opname_koorts: tab HighRiskPathogen

by FocusOnjuist_opname_koorts: tab Grampos

by FocusOnjuist_opname_koorts: tab Outpatientpresentation

by FocusOnjuist_opname_koorts: tab HA_BSI

by FocusOnjuist_opname_koorts: tab voorbehandeling

by FocusOnjuist_opname_koorts: tab VoorbehandelingAdequaat

by FocusOnjuist_opname_koorts: tab UWI

by FocusOnjuist_opname_koorts: tab GI

by FocusOnjuist_opname_koorts: tab PULM

by FocusOnjuist_opname_koorts: tab INTRAVASCULAIR

by FocusOnjuist_opname_koorts: tab SOFTTISSUE

by FocusOnjuist_opname_koorts: tab Other_Source

by FocusOnjuist_opname_koorts: tab FOCUSunidentified

by FocusOnjuist_opname_koorts: tab HighRiskSource

by FocusOnjuist_opname_koorts: tab AB_afgelopen2maanden

by FocusOnjuist_opname_koorts: tab Eerder_resistentie

by FocusOnjuist_opname_koorts: tab Gramnegresistentie6maanden

by FocusOnjuist_opname_koorts: tab ICMC6maanden

by FocusOnjuist_opname_koorts: tab Opname_nederland_6_maanden

by FocusOnjuist_opname_koorts: tab Chronische_urologische_ziekte

by FocusOnjuist_opname_koorts: tab VPH

by FocusOnjuist_opname_koorts: tab Immuungecompromiteerd

by FocusOnjuist_opname_koorts: tab Prednisolon

by FocusOnjuist_opname_koorts: tab DM

by FocusOnjuist_opname_koorts: tab Neutropenie

by FocusOnjuist_opname_koorts: tab Stamceltransplantatie

by FocusOnjuist_opname_koorts: tab Solid_Organ_transplantatie

by FocusOnjuist_opname_koorts: tab Livercirrhosis

by FocusOnjuist_opname_koorts: tab Malignancy

by FocusOnjuist_opname_koorts: tab Dialyse

by FocusOnjuist_opname_koorts: tab Fever

by FocusOnjuist_opname_koorts: tab Hypotensie

by FocusOnjuist_opname_koorts: tab Tachycardie

by FocusOnjuist_opname_koorts: tab Afwijkende_Neurologie

by FocusOnjuist_opname_koorts: tab Resistentie_huidigeBSI

by FocusOnjuist_opname_koorts: tab ICMC2dagen

by FocusOnjuist_opname_koorts: tab DezeresistentieBEKEND

by FocusOnjuist_opname_koorts: tab ziek

by FocusOnjuist_opname_koorts: tab Anaeroben

by FocusOnjuist_opname_koorts: tab sofamis

* Table 1 continuous variables

* If misdiagnosis ==1

drop if FocusOnjuist_opname_koorts==0

sum Age, detail

sum qsofa, detail

sum PITTscoreNew3, detail

* If misdiagnosis ==0

drop if FocusOnjuist_opname_koorts==1

sum Age, detail

sum qsofa, detail

sum PITTscoreNew3, detail

* Standardized mean differences

stddiff Age Sexe afname_uit_lijn Anaeroben HighRiskPathogen Grampos Outpatientpresentation HA_BSI voorbehandeling VoorbehandelingAdequaat UWI GI PULM INTRAVASCULAIR SOFTTISSUE Other_Source FOCUSunidentified HighRiskSource AB_afgelopen2maanden Eerder_resistentie Gramnegresistentie6maanden ICMC6maanden Opname_nederland_6_maanden Chronische_urologische_ziekte VPH Immuungecompromiteerd Prednisolon DM Neutropenie Stamceltransplantatie Solid_Organ_transplantatie Livercirrhosis Malignancy Dialyse Fever Hypotensie Tachycardie Afwijkende_Neurologie ziek PITTscoreNew3 qsofa Resistentie_huidigeBSI DezeresistentieBEKEND sofamis ICMC2dagen, by(FocusOnjuist_opname_koorts)

* Replace missing values, generate missing indicator

replace afname_uit_lijn=0 if afname_uit_lijn==.

gen sofamis = (qsofa ==.)

replace qsofa=0 if qsofa==.

* Propensity score model

psmatch2 FocusOnjuist_opname_koorts Age Sexe afname_uit_lijn HighRiskPathogen Grampos Outpatientpresentation HA_BSI voorbehandeling VoorbehandelingAdequaat UWI GI PULM INTRAVASCULAIR SOFTTISSUE Other_Source HighRiskSource AB_afgelopen2maanden Eerder_resistentie Gramnegresistentie6maanden ICMC6maanden Opname_nederland_6_maanden Chronische_urologische_ziekte VPH Immuungecompromiteerd Prednisolon DM Neutropenie Stamceltransplantatie Solid_Organ_transplantatie Livercirrhosis Malignancy Dialyse Fever Hypotensie Tachycardie Afwijkende_Neurologie PITTscoreNew3 qsofa Resistentie_huidigeBSI ICMC2dagen DezeresistentieBEKEND sofamis ziek Anaeroben, out (Mortaliteit14) caliper (0.02) noreplace neighbor(1) logit

* Check balance

pstest Age Sexe afname_uit_lijn HighRiskPathogen Grampos Outpatientpresentation HA_BSI voorbehandeling VoorbehandelingAdequaat UWI GI PULM INTRAVASCULAIR SOFTTISSUE Other_Source HighRiskSource AB_afgelopen2maanden Eerder_resistentie Gramnegresistentie6maanden ICMC6maanden Opname_nederland_6_maanden Chronische_urologische_ziekte VPH Centrale_Lijn Immuungecompromiteerd Prednisolon DM Neutropenie Stamceltransplantatie Solid_Organ_transplantatie Livercirrhosis Malignancy Dialyse Fever Hypotensie inotropie Tachycardie tube Afwijkende_Neurologie ziek PITTscoreNew3 qsofa Resistentie_huidigeBSI ICMC2dagen DezeresistentieBEKEND sofamis

* Distribution of propensity scores

kdensity _pscore if FocusOnjuist_opname_koorts==0, lwidth(medthick) addplot((kdensity _pscore if FocusOnjuist_opname_koorts==1, ///

lwidth(medthick))) xtitle(Propensity score) title(Propensity scores) legend(order(1 "" 2 ""))

* Save data

preserve

* Generate matched dataset

gen pair = _id if _treated==0

replace pair = _n1 if _treated==1

bysort pair: egen paircount = count(pair)

drop if paircount !=2

save paired, replace

* Table 2, see code for table 1 but use matched set and add sofamis (same for SMD)

* Figure 1

* Propensity scores of misdiagnosed patients

drop if FocusOnjuist_opname_koorts==0

sort _pscore

* Propensity scores of patients with correct diagnosis

drop if FocusOnjuist_opname_koorts==1

sort _pscore

* Back to original dataset

restore

* Varying caliper in propensity score matching

* Use previous code propensity score model and change caliper (0.2, 0.5)

** IPTW analyses

* IPTW weigths (for ATT)

gen IPWgewicht2 = FocusOnjuist_opname_koorts + ((1-FocusOnjuist_opname_koorts)*_pscore)/(1-_pscore)

sum IPWgewicht2

* Untruncated, unadjusted effect

reg Mortaliteit14 i.FocusOnjuist_opname_koorts [pw=IPWgewicht2],base

* Untruncated, adjusted effect (adjusted for solid organ transplantation, stem cell transplantation and presentation at outpatient clinicl before hospital admission)

reg Mortaliteit14 i.FocusOnjuist_opname_koorts Stamceltransplantatie Solid_Organ_transplantatie Outpatientpresentation [pw=IPWgewicht2],base

* IPTW, truncated >99th percentile

centile IPWgewicht2, centile(99)

scalar IPWgewicht2_99=r(c_1)

gen IPWgewichttrunc3=min(IPWgewicht2,IPWgewicht2_99) if IPWgewicht2 !=.

sum IPWgewicht2*

* Truncated >99th, unadjusted effect

reg Mortaliteit14 i.FocusOnjuist_opname_koorts [pw=IPWgewichttrunc3],base

* Truncated >99th percentile, adjusted effect

reg Mortaliteit14 i.FocusOnjuist_opname_koorts Stamceltransplantatie Solid_Organ_transplantatie Outpatientpresentation [pw=IPWgewichttrunc3],base

* IPTW, truncated >10

gen IPWgewichttrunc4=min(IPWgewicht2,10) if IPWgewicht2 !=.

sum IPWgewicht2*

* Truncated >10, unadjusted effect

reg Mortaliteit14 i.FocusOnjuist_opname_koorts [pw=IPWgewichttrunc4],base

* Truncated >10, adjusted effect

reg Mortaliteit14 i.FocusOnjuist_opname_koorts Stamceltransplantatie Solid_Organ_transplantatie Outpatientpresentation [pw=IPWgewichttrunc4],base

* Table 4, extremely high propensity scores

* New variable extremelyhighpropensity scores

drop if _pscore==.

generate extremelyhighpropensityscores= .

replace extremelyhighpropensityscores = 1 if _pscore>=0.95

replace extremelyhighpropensityscores = 0 if _pscore<0.95

replace extremelyhighpropensityscores = . if _pscore==.

* Table 4

sort extremelyhighpropensityscores

by extremelyhighpropensityscores: tab Sexe

by extremelyhighpropensityscores: tab afname_uit_lijn

by extremelyhighpropensityscores: tab HighRiskPathogen

by extremelyhighpropensityscores: tab Grampos

by extremelyhighpropensityscores: tab Outpatientpresentation

by extremelyhighpropensityscores: tab HA_BSI

by extremelyhighpropensityscores: tab voorbehandeling

by extremelyhighpropensityscores: tab VoorbehandelingAdequaat

by extremelyhighpropensityscores: tab UWI

by extremelyhighpropensityscores: tab GI

by extremelyhighpropensityscores: tab PULM

by extremelyhighpropensityscores: tab INTRAVASCULAIR

by extremelyhighpropensityscores: tab SOFTTISSUE

by extremelyhighpropensityscores: tab Other_Source

by extremelyhighpropensityscores: tab FOCUSunidentified

by extremelyhighpropensityscores: tab HighRiskSource

by extremelyhighpropensityscores: tab AB_afgelopen2maanden

by extremelyhighpropensityscores: tab Eerder_resistentie

by extremelyhighpropensityscores: tab Gramnegresistentie6maanden

by extremelyhighpropensityscores: tab ICMC6maanden

by extremelyhighpropensityscores: tab Opname_nederland_6_maanden

by extremelyhighpropensityscores: tab Chronische_urologische_ziekte

by extremelyhighpropensityscores: tab VPH

by extremelyhighpropensityscores: tab Immuungecompromiteerd

by extremelyhighpropensityscores: tab Prednisolon

by extremelyhighpropensityscores: tab DM

by extremelyhighpropensityscores: tab Neutropenie

by extremelyhighpropensityscores: tab Stamceltransplantatie

by extremelyhighpropensityscores: tab Solid_Organ_transplantatie

by extremelyhighpropensityscores: tab Livercirrhosis

by extremelyhighpropensityscores: tab Malignancy

by extremelyhighpropensityscores: tab Dialyse

by extremelyhighpropensityscores: tab Fever

by extremelyhighpropensityscores: tab Hypotensie

by extremelyhighpropensityscores: tab Tachycardie

by extremelyhighpropensityscores: tab Afwijkende_Neurologie

by extremelyhighpropensityscores: tab Resistentie_huidigeBSI

by extremelyhighpropensityscores: tab ICMC2dagen

by extremelyhighpropensityscores: tab DezeresistentieBEKEND

by extremelyhighpropensityscores: tab ziek

by extremelyhighpropensityscores: tab Anaeroben

by extremelyhighpropensityscores: tab sofamis

* Continuous variables

* table 4, extremelyhighpropensityscores ==0

drop if extremelyhighpropensityscores==1

sum Age, detail

sum qsofa, detail

sum PITTscoreNew3, detail

* table 4, extremelyhighpropensityscores==1

drop if extremelyhighpropensityscores==0

sum Age, detail

sum qsofa, detail

sum PITTscoreNew3, detail

***SMD table 4

stddiff Age Sexe afname_uit_lijn Anaeroben HighRiskPathogen Grampos Outpatientpresentation HA_BSI voorbehandeling VoorbehandelingAdequaat UWI GI PULM INTRAVASCULAIR SOFTTISSUE Other_Source FOCUSunidentified HighRiskSource AB_afgelopen2maanden Eerder_resistentie Gramnegresistentie6maanden ICMC6maanden Opname_nederland_6_maanden Chronische_urologische_ziekte VPH Immuungecompromiteerd Prednisolon DM Neutropenie Stamceltransplantatie Solid_Organ_transplantatie Livercirrhosis Malignancy Dialyse Fever Hypotensie Tachycardie Afwijkende_Neurologie ziek PITTscoreNew3 qsofa Resistentie_huidigeBSI DezeresistentieBEKEND sofamis ICMC2dagen, by(extremelyhighpropensityscores)
